# Factors influencing patients to choose one obesity treatment over another: a qualitative study

**DOI:** 10.1038/s41366-025-01789-3

**Published:** 2025-04-24

**Authors:** H. C. Craig, D. Alsaeed, E. Al Ozairi, S. Norris, J. Holian, C. Kennedy, H. Heneghan, D. McGillicuddy, E. Farrell, E. Hollmann, W. Al-Najim, C. W. le Roux

**Affiliations:** 1 Diabetes Complications Research Centre, UCD Conway Institute of Biomedical and Biomolecular Research, School of Medicine, University College Dublin, Belfield, Dublin 4 Ireland; 2 Dasman Diabetes Institute, PO Box 1180 Dasman, Kuwait; 3 Hepatology Department, St James’s Hospital, James’s Street, Dublin 8 Dublin, Ireland; 4 Nephrology Department, St Vincent’s University Hospital, Elm Park, Dublin 4 Dublin, Ireland; 5 Department of Pharmacology, St James’s Hospital and Clinical Research Facility, School of Medicine, Trinity College Dublin, Dublin, Ireland; 6 Surgery, School of Medicine, University College Dublin, Belfield, Dublin 4 Ireland; 7 School of Education, University College Dublin, Dublin, Ireland; 8 Department of Psychology, Maynooth University, Maynooth, Co, Kildare, Maynooth, Ireland

**Keywords:** Health policy, Obesity

## Abstract

**Background:**

When deciding a treatment option for the disease of obesity, knowing what the patient’s preferences are and what factors have influenced them can support healthcare professionals (HCPs) in tailoring their management plan. Little is known about patient preferences for obesity treatment. The present study was designed to identify factors that may influence these preferences.

**Objective:**

We aimed to identify factors affecting patient preferences and subsequent obesity treatment preferences among those seeking treatment for obesity-related disorders.

**Methods:**

Using a participatory action research design, we conducted World Café focus groups as part of a triangulation process with previously collected individual interviews and photovoice. Using purposive sampling, 12 patients with obesity complications of chronic kidney disease, type 2 diabetes, and metabolic dysfunction-associated steatotic liver disease took part. Thematic analysis was conducted to review themes, determine their significance, and report the findings.

**Results:**

Five themes emerged from the World Café on what influences patient treatment choice, which were 1) lack of resources, 2) healthcare professionals’ knowledge gaps, 3) lack of understanding, 4) emotional impact, and 5) support from family, friends, and HCPs. This data was then reviewed with the findings from the previous methodologies of one-to-one interviews and photovoice. Overall, four main themes were identified, which were 1) structural factors, 2) physical and emotional impact, 3) knowledge (of healthcare professionals and patients), and 4) support.

**Conclusion:**

This study demonstrates the importance of ensuring access to all treatment options by developing effective treatment plans with clear information for patients.

## Introduction

**Fig. 1:**
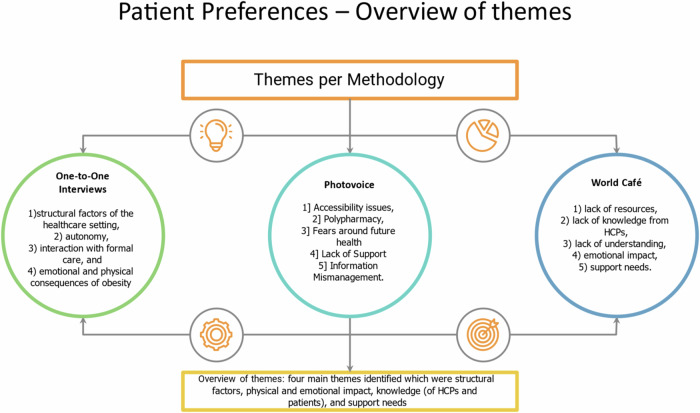
Emotional and Physical Complications that are challenging. Overview of themes.

Obesity is a global epidemic with a significant global impact on people and a substantial impact on health costs [1, 2]. The WHO found that 1 in 8 people worldwide will be living with obesity, and 2.5 billion adults (18 years and older) will be overweight [1]. Obesity is a gateway disease and increases a person’s chance of developing a non-communicable disease such as diabetes, chronic kidney disease, hypertension, and metabolic dysfunction-associated steatotic liver disease (MASLD) [3, 4]. Treatment options such as nutritional therapies, pharmacotherapies, and surgical therapies that produce >15% weight loss can make a significant health improvement for those with obesity complications [3]. As obesity is a complex, multifactorial disease, treatments should consider the patient’s health risk and metabolic profile [5]. To individualize treatment, both knowledge of the patient’s preferences and information about the specific physiology of a person is required. Given that at present we cannot predict an individual’s response to obesity treatments before starting the treatment, we think more focus should be on providing detailed information regarding the interventions to enable the patient to express a preference. The only way to find out what a patient wants is to ask them as part of the initial discussion. This helps patients to understand the risks and benefits of each option as well as manage their expectations [6]. Listening and responding to patient preferences regarding the choice of treatment in healthcare decision-making is necessary to incorporate shared decision-making into the practice of evidence-based medicine [7]. Thus, optimal treatment decisions in healthcare are informed not only by science but also by clinical expertise, taking into consideration patient preferences [6]. Shared decision-making in healthcare encourages collaboration between patients and clinicians, considering factors beyond clinical value, such as quality of life, to choose the most suitable treatment. However, patient preferences are not always definitive, and biases can affect both patients’ and clinicians’ decisions. A structured approach that integrates both parties’ values could lead to better outcomes by helping patients choose treatments aligned with their healthcare goals [8, 9]. This partnership has been shown to improve healthcare quality and outcomes, as it allows clinicians to understand patients’ concerns better. However, there is limited research on incorporating patient preferences, especially in complex cases like obesity complications. The lack of research in this area can lead to poor patient satisfaction, decreased adherence, and increased cognitive load on patients. The National Clinical Programme for Obesity’s adoption of clinical practice guidelines is a mechanism to change the focus of obesity to patient-centred care [10]. Patient-centred care means focusing on a patient’s particular healthcare needs [11]. The patient’s voice adds another perspective, and if the patient is not part of the process, it means that it is not patient-centred care [12]. This study represents the voice of patients with obesity-related complications. We aimed to triangulate data already collected using interviews and photovoice methodologies with new data using the World Café methodology to identify why patients select one obesity treatment over another. Figure 1 displays the different themes from each methodology.

## Methods

Using a participatory action research design, we conducted World Café focus groups as part of a triangulation process with previously collected individual interviews and photovoice. Using purposive sampling, 12 patients with obesity complications of chronic kidney disease, type 2 diabetes, and metabolic dysfunction-associated steatotic liver disease took part. Thematic analysis was conducted to review themes, determine the significance of themes, and report the findings. Ethical approval was obtained from the Human Research Ethics Committee- Sciences (HREC), University College Dublin, Ireland August 6^th^, 2021. There was no monetary incentive to participate in this study.

### Inclusion and exclusion criteria

Inclusion criteria included patients with obesity and complications of diabetes, liver disease, or kidney disease with a high BMI with BMI of 35 kg/m^2^. Inclusion criteria were males and females, 18–70 years old with obesity and complications such as diabetes, liver disease or kidney disease, and with informed consent. The exclusion criteria included people with severe dementia or Alzheimer’s disease and people who have no verbal communication.

#### World cafe recruitment

We contacted 50 patients attending clinics for obesity complications, including liver disease, chronic kidney disease, and hypertension, of whom 16 agreed to participate and 12 attended on the day. While the numbers were small, sufficient saturation of themes occurred to justify the number of participants as sufficient for this methodology. The 12 people who agreed to participate in World Café were recruited from the same clinics with diabetes or metabolic dysfunction-associated steatotic liver disease (MASLD) so they were representative of the 50 people contacted. Culturally, the majority of participants were Irish and white, however, many participants were of European descent and are now living in Ireland. Table 1 displays the characteristics of participants.

**Table 1:** Characteristics of participants.

Methodologies	Individual interviews		Photovoice		World Café	
Number of participants	33		10		12	
Sex	Female: 16	Male: 17	Female: 8	Male: 2	Female: 5	Male: 7
Media age	56 years		54 years		65 years	
Age range	18-75		18-75		18-73	
Average BMI	38 kg/m^2^		42 kg/m^2^		39 kg/m^2^	
Average BMI	37 kg/m^2^					

#### World Café study design and analysis

On the day of the World Café, participants joined a table of four to five other people living with obesity complications. There were three separate tables with one facilitator at each table. The facilitators were provided with a brief on their facilitation role and offered a notebook. A fourth facilitator also moved from group to group to listen and add additional comments.

**Fig. 2:**
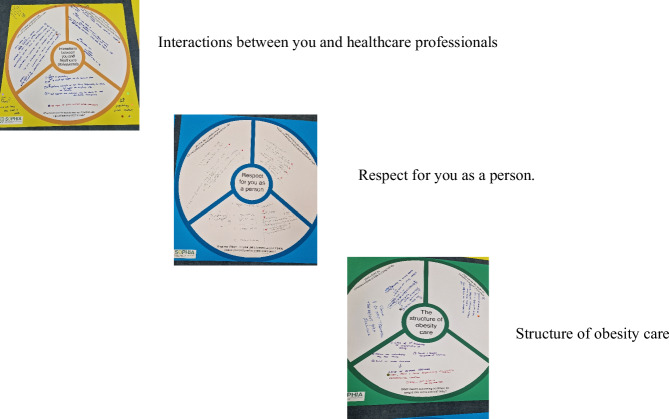
Emotional and Physical Complications that are manageable. Sample of World Café table mats.

**Fig. 3:**
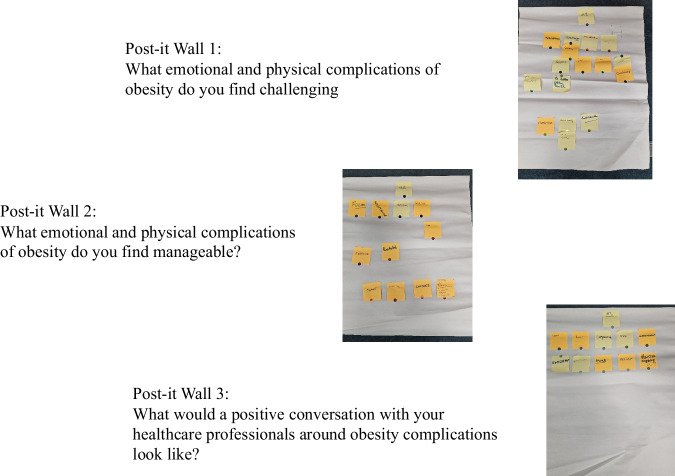
Conversation with Healthcare Professionals. Sample of post-it notes.

Before the table discussion began, the study, goals for each session, and ground rules of the World Café process were explained. Each table selected a note taker to place feedback on the specifically designed World Café table mats. A member of the table took notes on the discussion and placed them on the table mat, and another member of the group reported back on the discussion held after 20 min. One main question with three additional questions was placed on each table mat before each discussion. After each discussion, the table mats were removed and replaced with the next table mat, with the next question. After all the discussions, the table mats were photographed, and the findings were transcribed [see Fig. 2, Sample of Table mats]. Following the table mat discussions, one of the researchers introduced the Post-it note section, where participants were asked to identify one word to describe the challenges and successes of the physical and emotional impact of obesity. The facilitators assisted by collecting the notes and posting them on the wall. All participants then reflected on the notes presented and included additional comments [see Fig. 3, Sample of Post-it notes]. All participants contributed actively to the discussion and thus the ideas may have been initially generated by person A, but all the other then contributed meaningfully to deepen insight on these themes.

The researchers then photographed the paper sheets and Post-it notes. An iterative approach was used by the researchers to identify the themes, reviewing the codes to identify commonalities. Thematic analysis was conducted utilising Clarke and Braun’s (2013) six-step data analysis process, which is the familiarization of data, generation of codes, combining codes into themes, reviewing themes, determining the significance of themes, and reporting the findings. Two researchers developed a coding frame, which was uploaded to the data management software MAXQDA. Two researchers were involved in coding the transcripts; after the first coder completed coding, the second coder reviewed the transcript for validation. If there was any discrepancy in the coding of the transcripts, the two researchers with the PI discussed it to reach a consensus. The themes were thus developed by consensus and approved by the research team and the data were reviewed by the research team to ensure that multiple perspectives were considered [13]. Three reviewers determined data saturation; saturation was deemed to have occurred when the same themes and ideas were repeatedly emerging from the discussion as participants across the groups identified the same items (Table 2).

**Table 2:** Format, questions, and session prompts.

Questions and prompts
Theme 1: The Structure of Obesity Care had three questions for participants to discuss:
Do you think it is easy to access treatment for weight loss?
What makes accessing treatment for weight loss easier? Why?
What makes accessing treatment for weight loss more difficult? Why?
Theme 2: Respect for you as a person
Have you ever asked healthcare professionals for help to lose weight?
If not, why not?
What would/does limit your ability to exercise your right to choose your preferred treatment preference?
What would/does support you to exercise your right to choose your preferred treatment?
Theme 3: Interactions between you and the health care professionals
What support would you like to see if you were to seek treatment for weight loss?
Did your health care professional discuss management and or support options to help you lose weight? If not, why not?
What have been the experiences you have had with support from your HCP to date?
Post-it section: emotional and physical consequences of obesity
What emotional and physical complications of obesity do you find challenging?
What emotional and physical complications of obesity do you find manageable?
What would a positive conversation with your healthcare professional around obesity complications look like?

For this study, we used the qualitative results of previously collected individual interviews [14] and photovoice [15] While combining it with newly collected data from World Café, we used triangulation to explore what influences patients to prefer one obesity treatment over another. The methods and results of the one-to-one individual interviews and Photovoice can be found in our previously published work [14, 15]. World Café was the third methodology, enabling the researchers to learn the patients’ views, assess the saturation of themes, and represent the patients’ voices through participatory research. We chose the World Café method as it enabled people to share knowledge and perspectives about the treatment options for obesity complications. It is a useful method for exploring complex phenomena and engaging hard-to-reach participants. It helps participants gain multiple perspectives simultaneously and generate ideas as participants share knowledge and explore actions based on real-life experiences. The groups rotate on different discussion topics, giving everyone a chance to share their opinions and knowledge [16]. It is also a useful methodology to facilitate data triangulation using the data from other methods. In addition, this method assists in collecting rich qualitative data from multiple perspectives, encourages conversation and interaction among participants who may not interact otherwise, enhances trust and rapport creates a positive experience, and helps participants to work collaboratively in groups, learn from each other, and analyse and synthesize as a group to reach a consensus, and supports participatory evaluation and understanding in evaluation activities [16].

### Triangulation methodology

Triangulation in qualitative research is a technique used to enhance the credibility and validity of research findings. It involves the use of multiple methods, data sources, researchers, or theories to cross-check and confirm results. By examining a phenomenon from different angles, triangulation helps reduce bias, increase reliability, and provide a more comprehensive understanding of the research topic. The purpose of using more than one qualitative method before triangulation was to ensure that the research findings were not the result of the limitations or biases of any single method. For example, we previously completed interviews and photovoice to capture a broader perspective [14, 15]. Each method has its strengths and weaknesses, and by using multiple approaches, we can compensate for the weaknesses of any one method, leading to more robust and well-rounded conclusions. Thus, triangulation helps to strengthen the overall quality of the research and ensures that the findings are well-supported by diverse sources of data or perspectives. World Café was utilised as the third methodology to verify the data, and by using triangulation to gain a better understanding of the research problem. This enables data integration, maximizing each technique’s strengths and validating the research results [17]. Table 3 displays the triangulation of data results.

**Table 3:** Triangulation of data results.

Methodologies	Themes				
Interviews	Structural factors of the healthcare setting, such as access and cost	Autonomy, including lack of knowledge and the perception of not being heard	Interaction with formal care, experienced as a lack of knowledge and support	Emotional and physical consequences of obesity	
Photovoice	Lack of choice driven by cost and access	Lack of information about treatment options	The emotional and physical impact of obesity and its complications	Gaining knowledge of options	Impact of the COVID pandemic
World Cafe	Lack of resources	Healthcare professionals’ knowledge gaps	Lack of understanding	Emotional impact	Support from family, friends, and HCPs
**Overarching themes after triangulation of data**					
Structural factors	Physical and emotional impact	Knowledge (of healthcare professionals and patients)			Support

## Results

### World Café

World Café identified five main themes that affected patients’ choice for obesity treatments; these were limited access due to lack of resources, HCPs’ knowledge gaps, lack of understanding of the patients, the emotional impact of obesity, and support needs. [See Table 4: Patient perspectives of preferred treatment options for obesity complications]. These themes were similar to the other two methods of one-to-one interviews [14] and photovoice [15]. After triangulating the data for the three methodologies (individual interviews, Photovoice, and World Café), four main themes emerged around what impacts treatment decision-making. The themes were: structural factors (accessibility), the impact on the patient both physical and emotional, the depth of knowledge of their HCP and patients, and support. These themes identified factors that influenced patient’s decision-making to select one treatment above another. The work also identified the cost of obesity treatments, inaccessibility to community care, and lack of support as challenges for patients, which impact the management of their disease.

**Table 4:** Patient perspectives of preferred treatment options for obesity complications.

**Structure of obesity care**
Do you think it is easy to access treatment for weight loss?
Difficulty in accessing the correct treatment, money, and environment. Access to the right treatment – support feels robotic, not personalised.
Lack of resources – number of diabetics, long waiting list.
Stigmatised treatment
Lack of understanding of obesity and what factors lead to obesity, i.e. PCOS, environmental, doctors thinking it’s your fault.
Diagnosis of obesity by a GP never happens.
What makes accessing treatment for weight loss easier? Why?
Health care professionals need more education and training to facilitate access to obesity treatment for patients.
Reduction of stigma: allow people to talk and discuss that it’s not your fault.
Cost of treatment: private specialist consultant, weight loss in private clinics.
Better education of GPs – gently at school
Almost no services are available to the public unless you can afford private.
Medical research – volunteers get access that others should. /Online social media
More awareness – processed food
What makes accessing treatment for weight loss more difficult? Why?
Stigma
Lack of GP awareness of understanding of obesity.
Denial and delayed knowledge of the situation.
Focus on other conditions.
Lack of actual services
Cost–profit vs social responsibility.
GP not the answer – under pressure, lack of information
Confusion over +/− of treatments
Shame/Embarrassment
Lack of knowledge of the types of treatment
Solutions tend to be short-term.
Where to go to get treatment.
Cost of treatment
**Respect for you as a person**
Have you ever asked healthcare professionals for help to lose weight? If not, why not?
Yes – why – to improve health – intervention, address worry.
Waiting lists 8 years
Not heard – go lose weight.
Not initially, but yes, when health was impacted. Poor quality of life by then.
Public awareness of treatment is not through GP awareness.
Yes, for weight loss, not for obesity.
What would/does limit your ability to exercise your right to choose your preferred treatment?
Cost + knowledge
Supports and limits.
Aftercare – follow-up.
Availability – access to dietitian, psychological factors, stigma, embarrassment
Waiting times – accessibility. Access to care limits choice.
Social media influencers pose issues with misinformation.
Surgery tourism
Voice – afraid to speak, not heard, passive dismissal, stigma.
What would/does support you to exercise your right to choose your preferred treatment?
More availability
My choice – give a sense of control – safer – less scary.
Societal influence
Access to different treatments has to be requested rather than offered.
Knowledge of options from both the health care professional and the PWD.
Information/knowledge – doctors can’t recommend what they don’t know.
Access – cost/geographical
Campaigns – the mass public don’t know.
Education – knowing the susceptibility of other complications (stigma)
Group counselling treatment.
Public/Private sponsored
Designated – weight loss treatment centre/clinic.
**Interactions between you and healthcare professionals**
What support would you like to see if you were to seek treatment for weight loss?
Therapist
Dietician
GP/Consultant and physical aspect (physio/PT/Exercise/knowledge)
View all options available.
The doctor should present all the supports/options.
Continued support.
Better diagnosis
Clear picture of diagnosis and treatment
More compassion and education in the medical field (sensitive topic)
Motivational support
Obesity is understood, and adequate knowledge and recognition as a standalone disease.
Long-term care, follow-up, and continuity.
Holistic approach, not one treatment suits all.
Lack of finance forces decisions – no judgements!
Understand a person’s history – not the first attempt. Patient is heard, not pigeonhole.
Long-term treatment plan covering:
Nutrition
Exercise
Medical intervention
Motivation
Regular visits
Co-operation, collaboration between health areas
Empathy
Did your health care professional discuss management and or support options to help you lose weight? If not, why not?
Do it yourself? Limited advice
Doctors believe in limited options, no knowledge.
Others to help, referred to someone else.
Getting marginally better, GP dependent
No proper management was delegated to others.
Once the access system was progression
Overall disconnect between the health conditions discussed through others, not diagnosed with obesity.
Psychology is vital overall.
What have been the experiences you have had with support from your HCP to date?
Negative, resentful to go back, delivery brutal, looked down upon, no compassion.
Don’t care to help – sort it out yourself. GP overall good support, not for obesity at the start, no understanding.
GP stigmatising comments on not taking responsibility for obesity, but support for anything else. Not fantastic.
Good support for other conditions – only for obesity on the need for further investigations.
More support for other conditions rather than obesity.
What have been the experiences you have had with support from your HCP to date?
Hospital care – minimal basic information on the food pyramid
GP – no information

**Emotional and physical consequences of obesity complications**			
Post-it wall 1:			
What emotional and physical complications of obesity do you find challenging?			
Perception	Shame	Low Self-esteem	Lonely
Support	Time	Attitude	Clothes/Clothing
Physical Discomfort	To listen to other people	Exercising	Physical Activity/lack of fitness
Confidence			
Post-it wall 2:			
What emotional and physical complications of obesity do you find manageable?			
Focus	Acceptance	Appearance	Going Out
Exercise	Respectful	Size	Shame
Walking	Clothes	Emotional (has developed a hard shell to ignore adverse comments)	
Post-it wall 3:			
What would a positive conversation with your healthcare professional around obesity complications look like?			
Support	Respect	Compassion	Hope
Improvement	Roadmap	Practical	Mind
Relief	Positive empathy		

#### Limited access

The first theme related to a lack of resources manifests as a lack of access to treatment options. Long public hospital waiting lists before accessing care were used as an example to describe experiences of being stigmatised and a lack of understanding of obesity as a disease. Participants considered the lack of public services for patients with obesity as an example of the government prioritising monetary cost above social responsibility.

*‘Lack of actual services and lack of GP awareness of understanding of obesity’, Patient 1, Group 2*

Study participants described how they were self-conscious about seeking treatment, and that the long waiting lists did not help them choose treatments because once they decided to proceed with an option, the challenge was to try to access it. Moreover, long waits can lead to further weight gain and deteriorating mental health, which also affects their treatment choice as they are seeking more immediate ways to manage their health.

*‘Difficulty in getting access is frustrating and disheartening for people living with obesity.’Patient 2, Group 1*

The participants in World Café reported that the diagnosis of obesity by a general practitioner (GP) was very rare. Their experience was that GPs focused on obesity complications and not the disease of obesity itself. Participants in World Café stated they were stigmatised as there was still a belief that all patients have to do is eat less and move more. They also identified that the patients themselves have little awareness of obesity as a disease, and hence, this impacts the choice of treatment. It was reported that few services were available in the public system, forcing many patients to seek expensive private care, which impacts the choice of treatments. Participants expressed the view that the lack of public services for patients was about cost rather than care, even when treatments were shown to meet health economic thresholds for funding.

GPs were also perceived not to have the time or knowledge to treat obesity as a disease, which impacts treatment choice. They identified that there needs to be more awareness about obesity and research. Participants explained, from their perspective, that there was confusion around treatments available while the cost of treatments in the private sector was prohibitive, thus curtailing choices that may have been preferred.

#### Lack of understanding

Participants described there was limited advice from HCPs, and they had to ask other patients for information, which may impact their choice of treatment. Participants also described how they have to find information from social media influencers which can pose challenges with misinformation regarding the choice of medications. They felt that there was a need for more information campaigns, so the general public knows what is available. Stories of bariatric surgery tourism were highly publicised, but again, not having the full or correct information remains problematic when a choice of treatment needs to be made. Participants with obesity complications also described that they were afraid to speak and ask for information on treatments in case they were dismissed. Participants described how they needed to find out information about treatments themselves as they felt that HCPs could not recommend what they did not know. From patients’ perspectives, there needs to be more awareness about obesity, that it is a disease that can be treated, and it has to start in school.

*‘Lack of understanding of obesity and what factors lead to obesity i.e. PCOS, environmental, doctors thinking it’s your fault.’ Patient 3, Group 1*

#### Lack of knowledge of HCPs

Participants explained that they did try to speak to their HCPs about their preferences for treatments of obesity and to explore what interventions are possible. From their perspective, there were several barriers to obtaining treatment which included not being heard by the HCP, and no information or help being offered. The waiting lists for bariatric surgery in the public sector were very long and pharmacotherapy was only available privately. Often access to different treatments had to be requested it wasn’t offered, and patients had to do their research on treatment options. Participants felt that the age of the HCP had an effect, and that younger HCPs had more knowledge about treatment options. The participants in the study described that their HCP gave the view it was not their problem, and they only focused on advising on obesity complications such as the need to get their blood pressure or diabetes under control. It is important to note that a potential key contributory factor here was the lack of access HCPs had to treatments for their patients, therefore, they might have lacked the required experience to prescribe these treatments.

*“Information/knowledge – doctors can’t recommend what they don’t know’ Patient 4, Group 3*

*‘Health care professionals need more education and training to facilitate access to obesity treatment for the patient.’ Patient 5, Group 1*

#### Emotional impact of obesity

Participants in the study stated they would like to see a reduction of stigma in interactions they have with HCPs. Patients should be allowed to talk and discuss their disease and their treatment choices for obesity and its complications without being made to believe that it is all their fault. Participants described being stigmatised, citing that a lot of people do not think of medical or surgical treatments for obesity as options. They also expressed that they felt judged by HCPs when attempting to discuss their obesity and its complications, generating feelings of low self-worth and being self-conscious; this can lead to deterioration in mental health and avoidance of discussions regarding treatment options. The groups also described how they experienced stigmatising comments from their HCP; this was demonstrated by the HCP not taking responsibility for offering treatment for the disease of obesity and sometimes simply stating the patient needed to try harder. Participants experienced not being heard, fear of speaking up, being passively dismissed by HCPs, or being overly stigmatised, which all impacted treatment choice. Dismissal by their HCPs and lack of empathy made them feel like they were wasting the HCP’s time and stopped them from asking about treatment choices.

The Post-it wall was a method used to highlight the challenges around patients’ deciding and participating in obesity treatments, which included low self-esteem, loneliness, lack of fitness, and lack of confidence. The activities and emotional processes the patients in the study found manageable included focus, exercise, walking, and acceptance. When asked what a positive conversation with your HCP around obesity treatments would look like, they stated support, respect, compassion, hope, and empathy.

#### Support

Although community and family support were emphasised as important, participants described how support from HCPs around obesity treatments felt robotic and not personalised. They expressed that their HCP should present all available options and support to help them manage their chronic disease of obesity and its complications. Participants described that support for chronic treatment varies from HCP to HCP, however, all their experiences were the same in that support for obesity was never at the start of the process. They explained there was good support for the complications of obesity, but not obesity itself. Those who received good support managed their disease better. Participants explained that cooperation and collaboration between health areas would be helpful to supply support and thus enable them to make choices between obesity treatments. Their perspective was that empathy would provide much-needed support in the management of their disease.

### Experience of support from their HCP

*‘Negative, resentful to go back, delivery brutal, looked down upon, no compassion. Don’t care to help – sort it out yourself.’ Patient 6, Group 1*

## Discussion

Five themes emerged on factors that influence patients’ choice for specific treatments for obesity from the World Café which were 1) lack of resources, 2) lack of knowledge from HCPs, 3) lack of understanding, 4) emotional impact, and 5) support needs. The World Café results were consistent with the existing themes that emerged from the previous two study components of one-to-one interviews [14] and Photovoice [15]. The process of triangulation resulted in four main themes being identified which were structural factors, physical and emotional impact, knowledge (of HCPs and patients), and support needs.

Two significant sub-themes highlighted by participants in the World Café study were access to treatment options for obesity and the cost associated with treatment options. A major barrier was a lack of access to treatments for obesity such as nutritional therapy, pharmacotherapy, or surgical therapy. The lack of resources would often manifest as a sporadic referral to a dietitian. Participants who were considering bariatric surgery reported that HCPs would usually respond by suggesting they try harder or that they could try to have surgery abroad because the waiting time for surgery in the public system was exceptionally long. Having surgery privately was an option but the cost was often prohibitive. Pharmacotherapy was even more challenging to access. Patients without diabetes had to pay privately even if they had other complications from obesity. This prevented many participants from benefiting from this treatment. All the above are barriers experienced by patients trying to seek treatment.

In addition, the lack of consistency in care was not helpful, especially because by the time patients were asking their HCP for help, they had already tried many other options, often including commercial weight loss programmes. This resonates with recent research by Kim et al. who outlined barriers to obesity care including misinformation, insufficient training in obesity, and a lack of recognition of obesity as a disease and cost [18]. Cost was a major influencing factor. Rutstein et al. outlined that while cost-effective analysis is a useful tool for evaluating health-related interventions in resources-limited settings may violate the ethical principles of equity and distributive justice conflicting with societal values [17].

Patients may have different perspectives on what they want for their healthcare and their quality of life, and this can be influenced by their knowledge and the impact of their disease [19]. A lack of mobility, and deterioration in different complications such as diabetes, were key motivators to consider pharmacotherapy or surgery. Mobility particularly affected participants when attempting to go shopping, work, or do activities with their children. Obesity is a disease associated with a higher risk of anxiety and depression. Anxiety was repeatedly highlighted as impacting their quality of life, which motivated them to gain advice from their HCPs regarding treatment options to improve their health [20]. Furthermore, the constant medical appointments reminded patients that their lives were limited because of their obesity and its complications. From a patient’s perspective, it appeared that many HCPs had limited knowledge about obesity treatment options. This meant patients did not receive appropriate help to decide on treatment options [21, 22]. In addition, some participants experienced obesity stigma from the HCP which affected their engagement in seeking care. A lack of knowledge about treatment options as well as experiencing a lack of knowledge on the part of their HCPs was a barrier to the participants attempting to choose treatments.

Participants had difficulty accessing appropriate, curated information about the disease of obesity. This raised concerns about health literacy. Benjamin R et al. (2010) outlined that limited health literacy can affect the patient in several ways including challenges in explaining symptoms or to follow self-care instructions [23]. The European Health Literacy Survey (HLS-EU) conducted in eight countries: Austria, Bulgaria, Germany, Greece, Ireland, the Netherlands, Poland, and Spain found that 47% (1 in 2) of Europeans have limited levels of health literacy [24, 25]. They found that this has significant implications for patient health outcomes, health behaviours, equity of access to health services, health service utilisation, and public health expenditure [24, 25]. Bridging the gap between information received, understanding it, and how to implement it is essential for patients and requires HCPs to communicate and support better knowledge exchange [23]. There is a need for a strategy to address the knowledge gaps of HCPs at every level regarding treatments for their chronic diseases.

Another key influencing factor for participants was the vital need for support. Support from family, friends, community, and their HCPs enables better decision-making regarding treatment options. Patients discussed the challenges they had managing their disease and complications not just from a physical aspect, but an emotional one as well. Patients expressed the need for tangible psychosocial support and HCP support as two key areas that would facilitate decision-making regarding treatments [26]. Support works best when it is done collaboratively with the HCP and the patient [26]. Including a framework that allows the patient and HCPs to incorporate their values and beliefs can help establish the best course of action for each patient [8]. This involves partnerships between HCPs and patients with obesity and improves healthcare quality and outcomes [27]. The advantage is that the medical teams will know the wants and needs of the patients, thus helping them to understand the concerns of each patient [28]. This increases patients’ autonomy so that they can make treatment decisions best suited to them based on their values and needs [2]. While the concept of integrating patients’ wishes is just gaining momentum, there is currently limited research on how to best involve patients with obesity in evidence-based decision-making, particularly in terms of engaging patients in decision-making about treatment and interpretation of their health [28].

Taking patient preferences for treatment options into consideration when identifying their treatment plan might play an important role in the successful management of chronic disease in patients with obesity complications, as patients become more informed and have a sense of control over their health. Quality in healthcare involves reviewing patient experiences, satisfaction, and outcomes [29]. Integrating patients’ wishes into decision-making facilitates a patient-centred approach that can improve the effectiveness of interventions. This could be valuable for patients because this collaborative decision-making process might help all parties consider the factors that will enhance the quality of life for each patient as obesity affects a patient’s mental and physical well-being [29]. Patients with chronic diseases driven by obesity are almost a hidden group, as they attend multiple clinics managing multiple diseases driven by obesity. There is a deficit of information on patient’s preferences. This study looked at their treatment preference to represent their voice in what their experiences, satisfaction, and hopeful outcomes maybe if they had input in the treatment pathway discussion.

In our review of the literature, the themes identified such as knowledge, support, cost, and physical and emotional impacts all resonate with the views and experiences the patients expressed in our study. Patients with obesity complications are put into the chronic disease category and attend multiple clinics for these conditions but expressed there was no cohesion in their care especially if their conditions were driven by obesity. For example, cost was a recurring theme in the literature. In Kyle et al. cost was seen as a barrier, particularly with a younger age group of 44.7% compared with 20.7% of older adults [30] citing it as a main issue. Craig et al. found that cost was an important factor in influencing decision-making for treatments for intentional weight loss, especially considering choosing medication as this would be a lifelong treatment [31]. Some participants who were prescribed anti-obesity medications did not adhere to them as the participants couldn’t afford it [14]. Tang et al. (2018) reviewed the factors affecting the choice of bariatric surgery and found that those whose health insurance covered the cost had a higher rate of acceptance of bariatric surgery than those who had to pay out-of-pocket [32]. In addition, the cost was seen as a factor in healthy eating as Manning et al. found that the cost of more nutritious foods was also a barrier to participating in lifestyle interventions for treating obesity [33]. Discussing the practical side from monetary costs to care coordination might assist in establishing how to support patients’ preferences in treatment options [34].

This study indicates the importance of developing effective treatment plans with clear information and access to obesity treatments and support for chronic disease management to help patients better manage their disease. This has implications for health policy for ensuring more equitable care and patient engagement and the value of shared decision-making. Shared decision-making helps the patient and HCP decide the best course of action for the patient to improve their quality of life, and this may include several factors [6, 8]. The only way to identify what a patient want is to actively involve them in the initial discussions. Patients with obesity complications attend a variety of clinics but are often not attending an obesity clinic. The challenge for them, as they expressed it, was the lack of integration between each discipline. From their perspective, there was no coordination of services for patients with obesity complications. Good care was received from the designated clinics for an obesity-related complication, whereas obesity care was lacking.

When health policymakers decide on a policy, they engage with several stakeholders including the HCPs, patient groups, and other interested parties. In the case of obesity, they must direct the response to the epidemic to provide strategies for obesity control through community, health, workplace, and school interventions [35]. Obesity contributes to and, in some cases, causes other diseases such as diabetes, cancer, metabolic dysfunction-associated steatotic liver disease (MASLD), and kidney disease, which then involve a lot of health resources to manage them. Patients can become overwhelmed when dealing with multiple obesity-related complications such as diabetes. The challenge then becomes how health policymakers ‘empower’ the individuals and families if they do not know what the patient’s preferences would be, and how can policymakers identify preferences to allow empowerment in terms of interventions and treatment options. Gaining the perspectives of patients with obesity complications on treatment options enables us to represent their voices, enhancing patient-centred care. The concept of patient-centred care is a goal for most healthcare organisations. The development of public patient involvement (PPI) programmes and participatory action research has become an essential component to the development of quality health services. PPI is seen to inform health policy and ensure the system is patient-focused [36]. In addition, participatory action research (PAR) has several benefits from clinical, patient, and health policy perspectives as it can enable the development of new ideas and solutions. It can help inform changes in health management by optimising services for the benefit of patients and can provide equity by giving marginalised groups a voice and creating a greater level of access to policy development [36, 37].

Taking a rights-based approach to health policy advocates for more equity and more participation. True participation requires that all stakeholders, including non-state participants, are included in all areas of healthcare provision [38, 39]. As they are directly affected, patients with obesity complications are one of the key stakeholders to be considered in any health policy development. A rights-based approach is a framework that puts human rights at the forefront of any policy or process [40]. It is a mechanism designed to empower people to participate in decision-making. A rights-based approach to health policy advocates for more equity and more participation [38, 39]. The absence of knowing what choices for treatment options patients would prefer for obesity treatment can impede the development of effective treatment plans or indeed obesity health policy.

### Key recommendations

The European Commission has formally classified obesity as a chronic disease, consequently, it should now be viewed and treated like all other chronic diseases. Taking patient preferences into consideration when identifying their management plan facilitates patient engagement, plays an essential role in their adherence to treatment, and should result in improved treatment outcomes. Providing a structure that allows informed decision-making between the patient and the healthcare professional may help establish the optimal approach for patients. This can be done through 1) Education of health professionals to increase knowledge and improve support of health professionals on obesity and treatment planning strategies. 2) Improve patients’ health literacy by developing programs and information systems for patients to improve their knowledge of obesity and its complications. 3) Expand access to affordable obesity treatment to ensure equity and access to disease services and support for all citizens. 4) Create the legal framework and guidance to expand research on health care and promote opportunities to promote fairness, independence, dignity, respect, and equity through developing and enhancing patient-centred care systems in shared decision-making for patients with obesity. Future research on patient preferences on a larger scale from the state bodies would be important to gain further information that could benefit future care and provide key information for those trying to provide care for patients with obesity. Important to note that clinicians working in specialists’ obesity services often have an acquisition bias for example if they predominantly provide pharmacotherapy as part of their care they think all patients want pharmacotherapy, the same applies to specialist dieticians who provide high-quality nutritional therapies because often patients have already decided whether they will attend a specific service based on what is provided.

### Strengths and limitations

One key strength is that the findings are based on the lived experiences of the participants. In addition, building a rapport with the study participants allowed them to talk and have their opinions considered, small groups led to a relaxed environment that encouraged participation, as demonstrated by their quotes which contributed to the depth of the data. Several participants mentioned that partaking in the study has made them think about what they can do or change to make things better for themselves, which is consistent in other studies [41]. The participatory action research approach enabled participants to work in a group setting in a collaborative way to portray their voices. Developing the coding scheme and having two coders code the transcripts was a great strength especially because the second coder was a highly experienced qualitative researcher. Notwithstanding this, there were limitations including the small recruitment was particularly the case for the World Café method, because people must give a certain amount of time to participate, and some participants do not feel comfortable speaking in a group. While 50 participants were contacted, only 12 participants participated in the World Café; nevertheless, saturation was achieved which was determined as no new concepts or ideas emerged following the last discussion. Saturation was agreed upon with multiple researchers present at World Café.

An inherent limitation of all qualitative research is that the findings only pertain to those studied, albeit the emerging themes can be used in subsequent qualitative and quantitative research to better understand the research question. Another limitation is the views of HCPs are not known as these findings are the perspectives and views of the participants.

## Conclusion

Taking patient preferences for treatment options into consideration when identifying their treatment plan plays an important role in the successful management of chronic disease in patients with obesity complications. Integrating patients’ wishes into decision-making includes a patient-centred approach that can improve the effectiveness of interventions. This is valuable for patients with obesity challenges because this collaborative decision-making process helps all parties consider factors that improve each patient’s quality of life. After triangulating the data for all participants in each of the three methodologies of individual interviews, Photovoice, and World Café, four main themes emerged around what impacts decision-making for treatments. They were structural factors, physical and emotional impact, knowledge of HCP and patients, and support. Inaccessibility to care and lack of support are challenges for patients in managing their disease. This study demonstrates the importance of developing effective treatment plans with clear information, ensuring access to all options for obesity treatment options, and providing support to help people manage their disease.

## Data Availability

The datasets generated during and/or analysed during the current study are available from the corresponding author upon reasonable request.
